# CD36 deficiency inhibits proliferation by cell cycle control in skeletal muscle cells

**DOI:** 10.3389/fphys.2022.947325

**Published:** 2022-08-30

**Authors:** Jingyu Sun, Yajuan Su, Yaning Xu, Duran Qin, Qianhui He, Haiping Qiu, Jiatong Zhuo, Weida Li

**Affiliations:** Sports and Health Research Center, Frontier Science Center for Stem Cell Research, School of Life Sciences and Technology, Translational Medical Center for Stem Cell Therapy and Institute for Regenerative Medicine, Shanghai East Hospital, Tongji University, Shanghai, China

**Keywords:** CD36, proliferation, cell cycle, skeletal muscle cells, palmitic acid

## Abstract

Obesity-related muscular dysfunction and relative muscle atrophy affect an increasing number of people. Elucidating the molecular mechanisms of skeletal muscle cell development and growth may contribute to the maintenance of skeletal muscle mass in obesity. Fatty acid translocase (FAT/CD36), as a long-chain fatty acid transport protein, is crucial for lipid metabolism and signaling. CD36 is known to function in myogenic differentiation, and whether it affects the proliferation of skeletal muscle cells and the underlying mechanisms remain unclear. In this study, the effect of CD36 deficiency on skeletal muscle cell viability and proliferation was examined using C2C12 myoblasts. Results showed that the deletion of CD36 enhanced the inhibitory effect of PA on the proliferation and the promotion of apoptosis in skeletal muscle cells. Intriguingly, the silencing of CD36 suppressed cell proliferation by preventing the cell cycle from the G0/G1 phase to the S phase in a cyclin D1/CDK4-dependent manner. Overall, we demonstrated that CD36 was involved in skeletal muscle cell proliferation by cell cycle control, and these findings might facilitate the treatment of obesity-related muscle wasting.

## 1 Introduction

Obesity increases the risk of serious metabolic diseases, such as, type 2 diabetes, hyperlipidemia, cardiovascular disease, and even cancer (Saltiel and Olefsky, 2017). Besides the effects on metabolism, the function and mass of skeletal muscle are also affected by obesity (Teasdale et al., 2013). In patients with normal, moderate, and severe muscle mass decline, the prevalence rates of obesity are 6.1%, 81.8%, and 100%, respectively (Guida et al., 2019). Obesity-related muscular dysfunction and relative muscle atrophy affect an increasing number of people, and no therapy is available at present (Seebacher et al., 2017).

Skeletal muscle accounts for about 40% of human body weight and is one of the few organs that has a tremendous capacity for adaptation and regeneration. Skeletal myogenesis is regulated by the transcriptional control of various genes, including encoding myogenic regulatory factors (Zammit, 2017). Elucidating the molecular mechanisms of skeletal muscle cell development and growth contributes to provide new approaches to the treatment of obesity-related muscle dysfunction.

Palmitic acid (PA), as a saturated long-chain fatty acid (LCFA), is widely found in human food. PA concentrations are elevated in the blood of patients with obesity (Samuel et al., 2010). PA has been demonstrated to directly inhibit insulin signaling in hepatocytes and myotubes (Gao et al., 2010). In cultured cells, 0.4–1.0 mM PA or palmitate can trigger the models of insulin resistance (Charytoniuk et al., 2019). Fatty acid translocase (FAT/CD36), as a receptor and transporter for LCFA (Martin et al., 2011; Pepino et al., 2014), is engaged in various PA-mediated functions, such as lipid metabolism and signaling. A recent study reported that CD36 enhances vascular smooth muscle cell proliferation (Yue et al., 2019). However, knowledge on the regulation of skeletal muscle cell proliferation by CD36 under PA condition is lacking. Cell cycle is important for muscle cell proliferation. Thus, CD36, as a possible therapeutic strategy targeting various proteins of the skeletal muscle cell cycle or the upstream of the cell cycle, needs to be developed.

In this study, we first examined the effect of CD36 deletion on skeletal muscle cells proliferation and apoptosis. The RNA sequencing (RNA-Seq) technology is then manipulated to identify differentially expressed genes and enrich signaling pathways for the investigation of the potential mechanism of CD36 deficiency in this process. Finally, the critical influence of cyclin D1/cyclin-dependent kinase 4 (CDK4) is highlighted in CD36-regulated skeletal muscle cells cycle. The regulatory circuit of the CD36 gene that determines muscle cell proliferation should be further clarified and can be used for developing new therapeutics to treat obesity-associated muscle wasting.

## 2 Materials and methods

### 2.1 Cell culture and siRNA interference

High-glucose DMEM with 10% FBS (#10270-106, Thermo Fisher Scientific, Waltham, Massachusetts, United States) supplemented with L-glutamine (2 mmol/L) was used to culture C2C12 mouse skeletal muscle cells. siRNA duplexes targeting mouse CD36 (siCD36-1: 5′-GGA​UGA​CAA​CUU​CAC​AGU​UTT-3′; siCD36-2: 5′-GGA​UUG​GAG​UGG​UGA​UGU​UTT-3′), and the negative control (siCont, 5′-UUC​UCC​GAA​CGU​GUC​ACG​UTT-3′) were obtained from GenePharma. In accordance with the manufacturer’s protocol, siRNA with a final concentration of 20 nM was transfected into C2C12 cells using Lipofectamine RNAiMAX (#13778075, Thermo Fisher Scientific, Waltham, MA, United States). Western blot analysis validated the effectiveness of transfection.

### 2.2 Cell viability assay

For cell viability after PA treatment, C2C12 cells were cultured in 96-well plates at a density of 3000 cells/well, grown at 37°C overnight, treated with different concentrations of PA (0, 50, 100, 150, 200, and 300 μM) for 24 h, For cell viability after CD36 knockdown under BSA and PA condition, C2C12 cells were cultured in 96-well plates at a density of 6000 cells/well, grown at 37°C overnight and transfected with siCD36, then treated with PA (200 μM) for 24 h, added with 10 μl CCK8 solution (#40203ES60, Yeasen, Shanghai, China), and incubated for 4 h at 37°C. OD values at 450 nm were read by a microplate reader (SpectraMax iD3, Molecular Devices, Silicon Valley, United States).

### 2.3 Ki67 immunofluorescent staining

C2C12 cells were seeded into 24-well plates, interfered with siCont or siCD36, and treated with 200 μM PA. Paraformaldehyde (4%) was used for cell fixation at room temperature for 15 min, and 0.1% triton was punched at room temperature for 15 min. Donkey serum (5%) was used for blocking at room temperature for 2 h, and the primary antibody of Ki67 (dilution, 1:100, #MA5-14520, Thermo Fisher Scientific, Waltham, MA, United States) was used for incubation overnight at 4°C and washed thrice with PBST. The secondary antibody was incubated at room temperature for 1 h and washed three times with PBST, and slices were sealed with a water-soluble blocker containing DAPI (#36308ES11, Yeasen, Shanghai, China). The Zeiss confocal microscope (LSM880, Zeiss, Oberkochen, Germany) was used for image observation in acquisition, and Ki67 positive cells were counted by Image J software (V1.8.0.112) and calibrated with DAPI.

### 2.4 Flow cytometry analysis

#### 2.4.1 Cell cycle assays

Flow cytometry analysis of the cell cycle was conducted by treating C2C12 cells with 200 μM PA after siCD36 interference. Then, cells were harvested and washed thrice in PBS. Single-cell suspension was prepared by digesting cells with trypsin, centrifugation for 3–5 min at 1000 g, and precipitating the cells. Wash the cells with pre-chilled PBS and centrifuge again. For cell fixation, resuspend the pellets with 1 ml of pre-chilled 70% ethanol at 4°C for 2 h. An appropriate amount of propidium iodide (PI) staining solution was prepared in accordance with the cell cycle assay kit (#C1052, Beyotime Biotechnology, Shanghai, China). Cells were resuspended with 500 μL PI staining solution per tube, incubated for 30 min at 37°C, protected from light, and subjected to BD flow cytometry (FACSVerse, BD Biosciences, NY, United States) at the excitation wavelength of 488 nm for red fluorescence and light scattering at the same time. The cell cycle percentage was analysed using the FlowJo V10 software (V10.0.7).

#### 2.4.2 Apoptosis assays

For the apoptosis test, cells were seeded at a density of 3 × 10^4^ cells per dish before siRNA interference, treated with 200 μM PA for 4 h to induce apoptosis, washed twice with PBS, and digested by trypsin into a single-cell suspension. 5 μl Annexin V–FITC and 10 μl PI (#C1062, Beyotime Biotechnology, Shanghai, China) were used to label cells apoptosis, incubated for 10–20 min at room temperature, protected from light, and rinsed once with PBS. The percentage of apoptotic cells was detected by flow cytometry (FACSVerse, BD Biosciences, NY, United States). Cells without staining were used as negative control. These FACS data were analyzed by the FlowJo V10 (V10.0.7).

### 2.5 Transcriptome sequencing

#### 2.5.1 RNA extraction, cDNA library construction and RNA-sequencing

The TRIzol reagent (Invitrogen, Carlsbad, CA, United States) was used to isolate and purify total RNA in accordance with the manufacturer’s instructions. The NanoDrop ND-1000 was used to determine the amount and purity of RNA in each sample (NanoDrop, Wilmington, DE, United States). The Agilent 2100 with RIN number > 7.0 was used to assess RNA integrity.

Two rounds of purification were used to extract poly (A) RNA from total RNA (5 µg) by using poly-T oligo-attached magnetic beads. The poly(A) RNA was then broken into little fragments under high temperature by using divalent cations. Cleaved RNA fragments were then reverse-transcribed into cDNA, which was then utilized to make U-labeled second-stranded DNAs by using *E. coli* DNA polymerase I, RNase H, and dUTP. The blunt ends of each strand were treated with an A-base to prepare them for ligation to the indexed adapters. The T-base overhang on each adapter was used to ligate the adaptor to the A-tailed fragmented DNA. Single- or dual-index adapters were ligated to fragments, and size selection was performed with AMPureXP beads after the heat-labile UDG enzyme treatment of the U-labeled second-stranded DNAs. Ligated products were amplified with PCR. The resultant cDNA library had an average insert size of 300 bp (± 50 bp). A cDNA library constructed by Illumina NovaseqTM 6000 sequence platform (LC Bio, Hangzhou, China). Using the Illumina paired-end RNA- sequencing approach, we sequenced the transcriptome, generating a total of million 2 × 150 bp paired-end reads.

#### 2.5.2 Sequence analysis

Reads from all samples were compared to the reference genome using the HISAT2 software package. HISAT2 builds a database of potential splice junctions and confirms these by comparing the previously unmapped reads against the database of putative junctions. The mapped reads of each sample were assembled using StringTie. Then, all transcriptomes from samples were merged to reconstruct a comprehensive transcriptome using perl scripts. After the final transcriptome was generated, StringTie and edgeR were used to estimate the expression levels of all transcripts. StringTie was used to perform expression level for mRNAs by calculating FPKM.

#### 2.5.3 Differentially expressed genes analysis

Genes differential expression analysis was performed by edgeR software between two different groups. The genes with *p* value < 0.05 and absolute log2 (fc) ≥ 1 were considered differentially expressed genes. Differentially expressed genes were then subjected to enrichment analysis of KEGG pathways. The raw data were submitted to the National Center for Biotechnology Information (NCBI) Sequence Read Archive (SRA) database under accession numbers: GSE204686.

#### 2.5.4 KEGG pathway analyses

Pathway enrichment analysis was performed mainly using annotated differential genes as foreground genes and all annotated genes on the genome as background genes. The significantly enriched KEGG pathways (*p* < 0.05) was defined using hypergeometric test.

### 2.6 Real-time quantitative PCR

Total RNA was isolated using TRIzol (Invitrogen, Carlsbad, CA, United States) reagent. The RNA was fully released after 5 min at room temperature and added with 100 μl chloroform. Centrifuge at 12000 r for 15 min, take about 160 μl of the upper aqueous phase, add an equal amount of isopropanol, and mix them thoroughly. The RNA precipitate was washed twice with 75% ethanol and dissolved in 20 μl RNase-free water, and the RNA concentration was measured by NanoDrop. The 260/280 ratio was used to determine the RNA quality. The cDNA was obtained by taking 1 μg total RNA and reverse transcription in accordance with the instructions of cDNA synthesis kit (#KR106-02, TIANGEN, Beijing, China). The reaction system of real-time fluorescence PCR was as follows: 1 μl cDNA template, 5 μl 2×FastStart Universal SYBR GreenMaster (#FP205-02, TIANGEN, Beijing, China), 0.5 μl each of forward and reverse primers, and 4 μl RNAse-free water. The sequence of all primers are shown in Table 1. The PCR reaction procedure was as follows: predenaturation at 95°C for 10 min; product amplification cycle reaction at 95°C for 10 s; and heating at 60°C for 10 s and 72°C for 10 s for 40 cycles. The total RNA quantity of the samples was calibrated using GAPDH as an internal reference, and the RNA expression level of the target gene was calculated using the 2^−ΔΔCt^ method.

**TABLE 1:** Sequences of primers for qRT-PCR.

Gene	Sense (5′–3′)	Anti-sense (5′–3′)
Myf-5	AAG​GCT​CCT​GTA​TCC​CCT​CAC	TGA​CCT​TCT​TCA​GGC​GTC​TAC
MyoD	CCA​CTC​CGG​GAC​ATA​GAC​TTG	AAA​AGC​GCA​GGT​CTG​GTG​AG
MRF4	AGA​GGG​CTC​TCC​TTT​GTA​TCC	CTG​CTT​TCC​GAC​GAT​CTG​TGG
Myogenin	GAG​ACA​TCC​CCC​TAT​TTC​TAC​CA	GCT​CAG​TCC​GCT​CAT​AGC​C
Mcm5	CAG​AGG​CGA​TTC​AAG​GAG​TTC	CGA​TCC​AGT​ATT​CAC​CCA​GGT
Gsk3b	TGG​CAG​CAA​GGT​AAC​CAC​AG	CGG​TTC​TTA​AAT​CGC​TTG​TCC​TG
Bub1	AGA​ATG​CTC​TGT​CAG​CTC​ATC​T	TGT​CTT​CAC​TAA​CCC​ACT​GCT
Ccna2	AAG​AGA​ATG​TCA​ACC​CCG​AAA​AA	ACC​CGT​CGA​GTC​TTG​AGC​TT
Orc1	ATT​CAC​ATC​AAG​GTT​GGA​CAG​TT	CGG​CCT​AAC​AAG​TGC​CTT​T
Cdkn1c	CGA​GGA​GCA​GGA​CGA​GAA​TC	GAA​GAA​GTC​GTT​CGC​ATT​GGC
Ttk	GCA​GTG​TGA​CGA​TTG​ATT​CCA	TCG​GCA​CAG​ATT​TTA​GAC​AAG​C
Skp2	ATG​GAC​TGC​TCT​CAA​ACC​TCG	CCT​GGA​AAG​TTC​TCC​CGA​CTA​A

### 2.7 Western blot analysis

The RIPA buffer (1% Nonidet P-40, 50 mM Tris-HCl, 0.5% sodium deoxycholate, 150 mM NaCl, and 0.1% SDS) with cocktail (Roche Diagnostics, Germany) was used to lyse the cells. Protein concentrations were determined using the BCA assay (Thermo Scientific, Waltham, MA, United States). The loaded proteins were separated using SDS-PAGE and then transferred to polyvinylidene difluoride membranes (Millipore Corp., Bedford, MA, United States). After incubation with blocking buffer (5% non-fat milk solution) for 1 h, membranes were incubated with the following primary antibodies: rabbit antibody cleaved caspase-3 (Asp175) (#9661), cyclin D1 (#2978T), cyclin E1 (#4129), and CDK4 (#12790T) purchased from Cell Signaling (Danvers, MA, United States), goat antibody anticluster of differentiation 36 (CD36, #AF2519) from R&D system (Minneapolis, MN, United States), and glyceraldehyde-3-phosphate dehydrogenase (GAPDH, #AB0037) from Abways (Shanghai, China). After incubation with peroxidase-conjugated secondary antibodies (Yeasen, Shanghai, China) for 1 h, membranes were rinsed thrice. Target protein bands were detected by the ECL luminescence method, and bands were analyzed using the Image J software for grayscale values.

### 2.8 Statistical analysis

Data were expressed as mean ± SEM. For multiple comparisons, statistical significance was established using an unpaired Student t-test or a one-way ANOVA The significance level was set to *p* < 0.05. The SPSS 19.0 (Chicago, IL, United States) was used to analyze the data.

## 3 Results

### 3.1 CD36 deficiency inhibits the proliferation of C2C12 myoblasts

**FIGURE 1:**
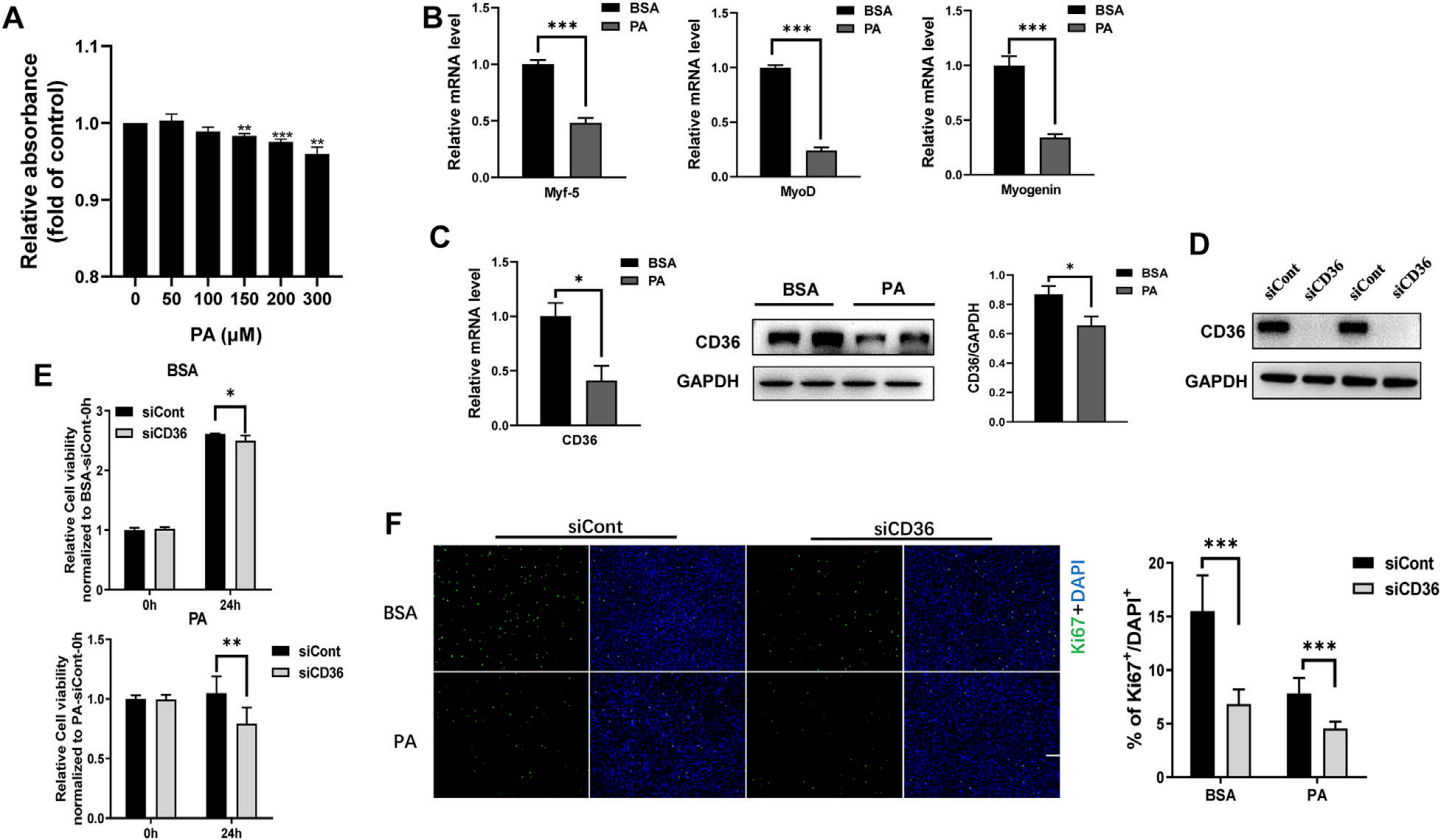
Knockdown of CD36 inhibits the proliferation of C2C12 myoblasts. **(A)** Effects of PA (0, 50, 100, 150, 200, and 300 μM) on C2C12 myoblast proliferation determined using the CCK8 analysis. **(B)** RT-PCR analysis of myogenic factor expressions (Myf5, MyoD, and myogenin) in C2C12 myoblasts after PA treatment. **(C)** RT-PCR, western blot analysis and quantification of CD36 expression in C2C12 myoblasts after PA treatment. **(D)** Western blot analysis of CD36 after CD36 knockdown by CD36 siRNA. **(E)** Effect of PA and/or CD36 siRNA on C2C12 myoblast viability determined using the CCK8 analysis. **(F)** C2C12 myoblasts stained with Ki67 after siCont or siCD36 transfection (scale bar = 200 μm). Values were presented as mean ± SEM of three independent experiments. *p < 0.05, **p < 0.01, ***p < 0.001 vs. the siCont group.

Obesity affects the function and mass of skeletal muscle, leading to a relative reduction in functional skeletal muscle mass. Thus, elevated circulating free fatty acid levels and ingested saturated fatty acids in individuals with obesity are hypothesized to inhibit skeletal muscle cell proliferation. PA is a major circulating saturated FFA and accounts for 30%–40% of serum FFAs (Pilz and März, 2008). Therefore, PA was used to mimic the hyperlipidemic environment of patients with obesity *in vitro* to study the effects of obesity on the proliferation of skeletal muscle cells. C2C12 myoblasts were treated with different concentrations (0, 50, 100, 150, 200 and 300 μM) of PA for 24 h. Cell proliferation was detected by CCK8 method, and the results showed that PA inhibited the proliferation of C2C12 myoblasts in a dose-dependent manner (Figure 1A). To further explore the effect of PA on myogenic cell differentiation, we examined the expression levels of myogenic factors. Our results showed that PA induced a decrease in the mRNA expression levels of Myf5, MyoD and myogenin (*p* < 0.001; Figure 1B), which is consistent with other study (Nakamura et al., 2021). Interestingly, both mRNA and protein expression levels of CD36 were decreased in C2C12 myoblasts after PA treatment, suggesting that CD36 expression levels may be involved in the process of skeletal muscle cell proliferation and differentiation (*p* < 0.05; Figure 1C). It is reported that CD36 plays an important role in cell fusion during myogenic differentiation (Park et al., 2012), but the role of CD36 on myogenic cell proliferation is not well understood. Therefore, the present study focused on the effect of CD36 on cell proliferation.

Some studies reported that CD36 affects the proliferation of vascular smooth muscle cells (Schlich et al., 2015), but whether it affects the proliferation of skeletal muscle cells remains unclear. In this study, CD36 expression was knocked down with CD36 siRNA to determine whether CD36 was engaged in the proliferation of C2C12 myoblasts. CD36 protein expression was reduced remarkably by CD36 siRNA (Figure 1D). Furthermore, CD36 deletion significantly decreased cell viability under BSA and PA conditions (*p* < 0.01, *p* < 0.05; Figure 1E), which indicated that the presence of CD36 was essential for cell proliferation. To further examine the effect of CD36 deletion on skeletal muscle cell proliferation, we used cellular immunofluorescence staining to label ki67. Results showed that the proliferative capacity of CD36-deficient C2C12 myoblasts was significantly reduced under BSA and PA conditions compared to the control group (*p* < 0.001; Figure 1F).

### 3.2 CD36 deficiency promotes palmitic acid-induced apoptosis in C2C12 myoblasts

**FIGURE 2:**
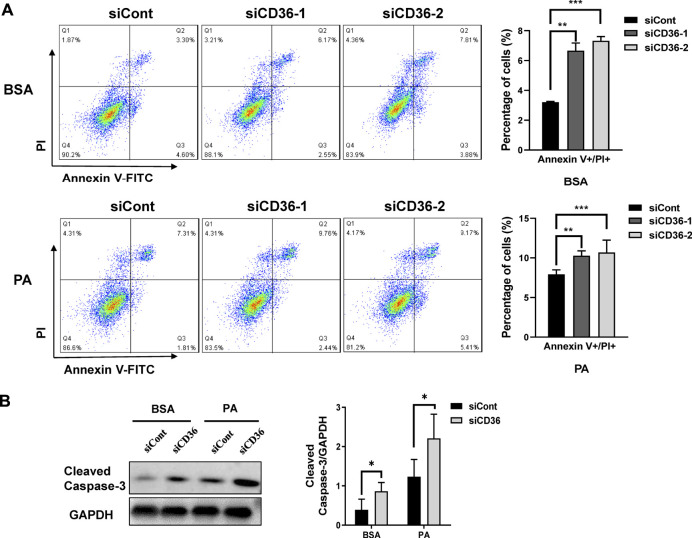
CD36 deficiency promotes PA-induced apoptosis in skeletal muscle cells. **(A)** Representative images of apoptosis in C2C12 myoblasts after siCont or siCD36 transfection under the conditions of BSA and PA treatments. Cell populations of different status were calculated. **(B)** Left: western blot analysis of cleaved caspase-3 in C2C12 myoblasts after siCont or siCD36 transfection in response to BSA or PA treatment; right: the quantification of cleaved caspase-3. Values are expressed as mean ± SEM of three independent experiments. *p < 0.05, **p < 0.01, ***p < 0.001 vs. the siCont group.

The number of viable skeletal muscle cells depends on proliferation and apoptosis. Results in Figure 1 demonstrated that CD36 deletion reduced the proliferation of C2C12 cells. Therefore, the effect of CD36 deletion on skeletal muscle cell apoptosis was further examined using flow cytometry. Our results showed that the late apoptotic cell population was increased by 2.9%–4.5% in the CD36 knockdown group (siCD36-1, siCD36-2) compared with that in the control group (siCont) under BSA conditions (*p* < 0.01, *p* < 0.001; Figure 2A). The CD36 knockdown group (siCD36-1, siCD36-2) had increased late apoptotic cell population by 1.9%–2.5% and significantly reduced live cell population compared with the control group (siCont) under PA conditions (*p* < 0.01, *p* < 0.001; Figure 2A). The expression levels of cleaved caspase-3 were elevated in the CD36 knockdown group under BSA and PA conditions compared to the control group (*p* < 0.05; Figure 2B). These results indicated that CD36 deletion remarkably induced apoptosis in skeletal muscle cells. Overall, the significant reduction in the cell viability of CD36 deficient cells was due to the combined effect of reduced cell proliferation and increased apoptosis.

### 3.3 CD36 deficiency upregulates cell cycle-related gene expression levels in C2C12 myoblasts

**FIGURE 3:**
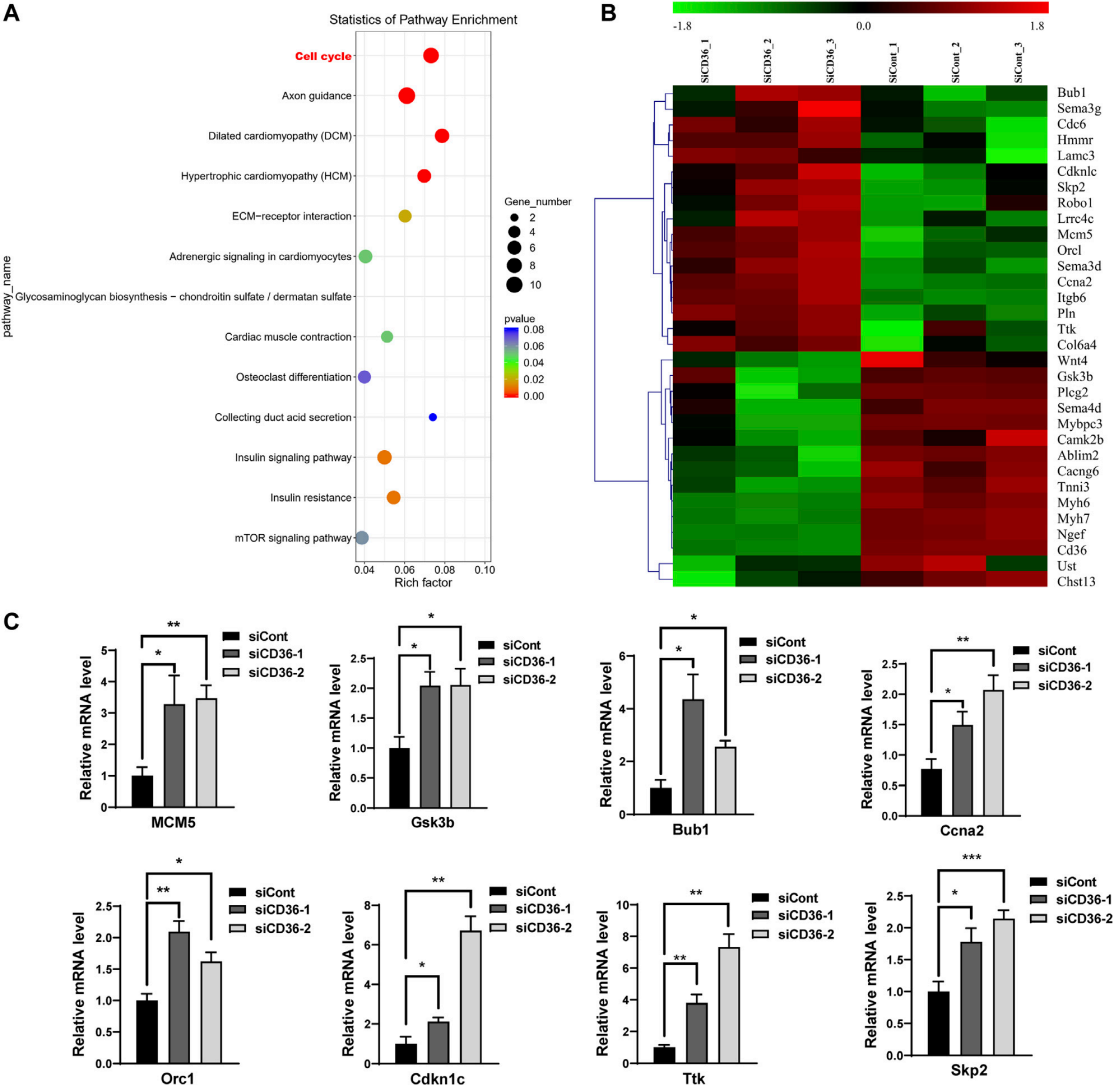
Differences in the gene expression in C2C12 myoblasts between siCont and siCD36 groups. **(A)** KEGG pathway analysis of DEG in C2C12 myoblasts after transfection with siCont and siCD36. **(B)** Hierarchical clustering analyses were performed based on all DEG in C2C12 myoblasts after transfection with siCont or siCD36. **(C)** qRT-PCR analysis of representative cell cycle-related gene expression in C2C12 myoblasts after transfection with siCont or siCD36. Each gene expression level was normalized to that of the GAPDH gene. Values are expressed as mean ± SEM of three independent experiments. *p < 0.05, **p < 0.01, ***p < 0.001 vs. the siCont group.

To further investigate the potential mechanism of cell proliferation inhibition by CD36 knockdown, we performed whole-genome transcript-level sequencing of CD36 knockdown cell lines. 521 differentially expressed genes were identified (Supplementary Table S1). The function of the known DEG was examined by KEGG enrichment analysis (Figure 3A). Some of the enriched pathways were related to muscle development and contraction, including cell cycle, adrenergic signaling in cardiomyocytes, cardiac muscle contraction, dilated cardiomyopathy (DCM), and hypertrophic cardiomyopathy (HCM). Many of the pathways were involved in glucose metabolism, including insulin signaling pathway and insulin resistance. In addition, three enriched pathways were related to cell junction, including extracellular matrix (ECM)-receptor interaction, axon guidance, and glycosaminoglycan biosynthesis - chondroitin sulfate/dermatan sulfate. The enrichment scatter plot below shows the differentially expressed genes’ enriched pathways. DEG were investigated using hierarchical clustering analysis (Figure 3B). Of the 31 DEG related to muscle development and contraction, 8 DEG were related to muscle cell cycle (Table 2), indicating that CD36 is critical to the muscle cell cycle. 13 DEG were related to muscle hypertrophy, 6 DEG were related to muscle remodelling, and 4 DEG were related to muscle contraction, suggesting that CD36 may have an important regulatory role in muscle gene transcriptions. To explore the effects of CD36 deletion on muscle cell cycle, transcript abundance of 8 key genes involved in cell cycle was quantified by qRT-PCR. Compared with RNA-seq results, the expression of cell cycle-related genes (i.e., MCM5, Gsk3b, Bub1, Ccna2, Orc1, Cdkn1c, Ttk, and Skp2) were further confirmed upregulation (Figure 3C). Together, these results suggested that the upregulation of cell cycle-related gene expression after CD36 knockdown might be an important mechanism to inhibit cell proliferation.

**TABLE 2:** Analysis of differentially expressed genes in cell cycle pathway between siCont and siCD36 groups by RNA-Seq.

Pathway_name	Gene_name	Gene_ID	Description	FC	log2(FC)	*p* Value	Regulation
Cell cycle	Mcm5	ENSMUSG00000005410	minichromosome maintenance complex component 5	2.05	1.04	0.00	up
Cell cycle	Cdc6	ENSMUSG00000017499	cell division cycle 6	2.45	1.30	0.00	up
Cell cycle	Bub1	ENSMUSG00000027379	Budding Uninhibited By Benzimidazoles 1	2.73	1.45	0.00	up
Cell cycle	Ccna2	ENSMUSG00000027715	cyclin A2	2.08	1.05	0.00	up
Cell cycle	Orc1	ENSMUSG00000028587	origin recognition complex, subunit 1	4.30	2.10	0.00	up
Cell cycle	Cdkn1c	ENSMUSG00000037664	cyclin-dependent kinase inhibitor 1C	2.94	1.56	0.00	up
Cell cycle	Ttk	ENSMUSG00000038379	Ttk protein kinase	2.16	1.11	0.00	up
Cell cycle	Skp2	ENSMUSG00000054115	S phase kinase-associated protein 2	2.00	1.00	0.00	up

### 3.4 CD36 deficiency induces cell cycle arrest in the G0/G1 phase of C2C12 myoblasts

**FIGURE 4:**
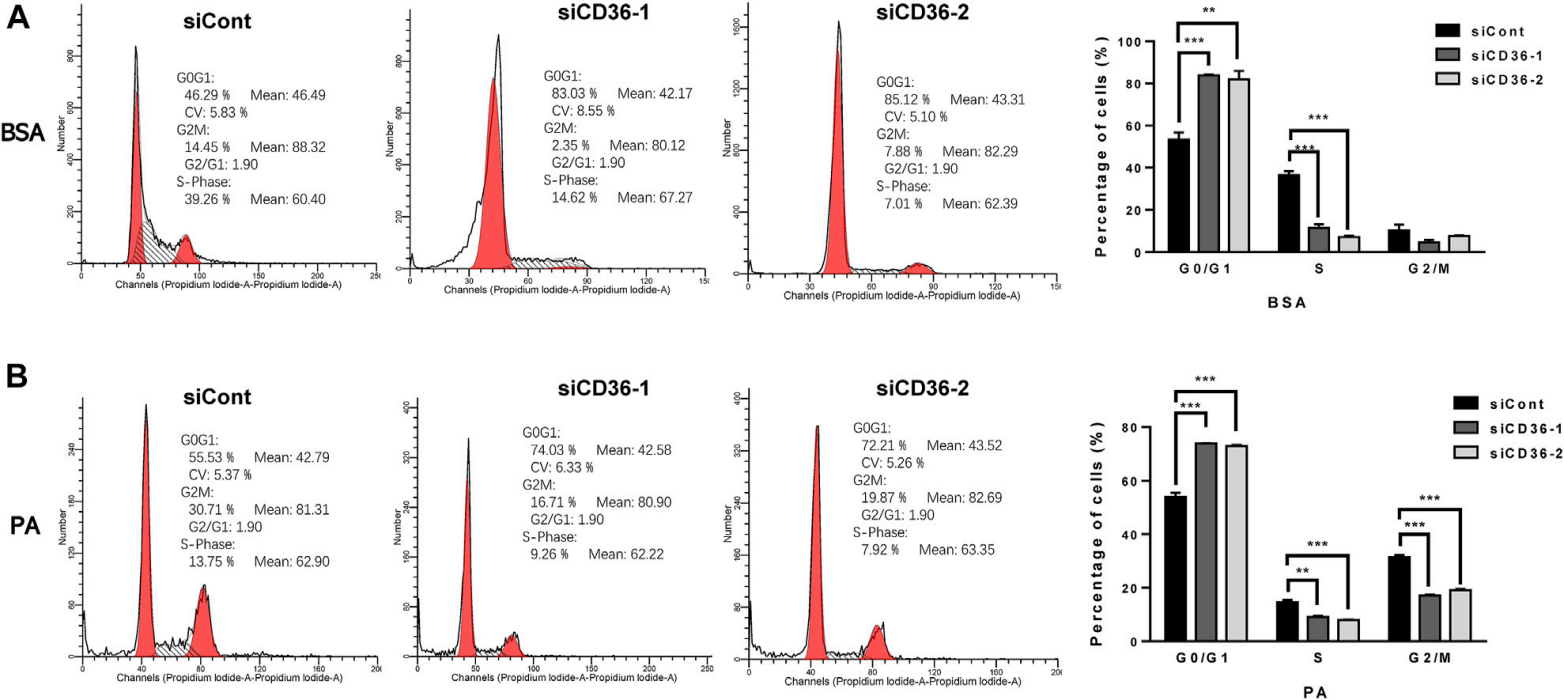
Induced C2C12 muscle cell cycle arrest in the G0/G1 phase by CD36 deficiency. **(A)** Proportion of C2C12 myoblasts in different cell cycle stages after siCont or siCD36 transfection under the condition of BSA treatment. The two prominent peaks (in red) represent G0/G1 and G2/M phase cells, respectively. The intermediate region between peaks (black hashes) represents S phase cells. **(B)** Proportion of C2C12 myoblasts in different cell cycle stages after siCont or siCD36 transfection under the condition of PA treatment. The two prominent peaks (in red) represent G0/G1 and G2/M phase cells, respectively. The intermediate region between peaks (black hashes) represents S phase cells. Values are expressed as mean ± SEM of three independent experiments. **p < 0.01, ***p < 0.001 vs. siCont.

As described in the high-throughput sequencing results, CD36 knockdown resulted in the upregulation of cell cycle-related gene expression levels (Figure 3). However, the effects of CD36 deletion on the skeletal muscle cell cycle is unclear. We investigated the effect of CD36 deficiency in cell cycle progression by using flow cytometry based on PI staining to learn how CD36 deficiency affected C2C12 myoblasts proliferation. Under BSA conditions, flow cytometry results showed that downregulation of the CD36 gene in skeletal muscle cells resulted in a significant increase in the percentage of cells in G0/G1 phase (29%–31%) and a corresponding decrease in the percentage of cells in S phase (25%–29%) (*p* < 0.01, *p* < 0.001; Figure 4A). In addition, the inhibition of CD36 expression under PA conditions significantly increased the percentage of cells in the G0/G1 phase (19%–20%), correspondingly decreased the percentage of cells in the S phase (6%–7%) in skeletal myocytes, and significantly decreased their percentages in the G2/M phase (*p* < 0.01, *p* < 0.001; Figure 4B). These findings implied that CD36 deficiency caused cell cycle arrest in the G0/G1 phase after CD36 silencing under BSA and PA conditions.

### 3.5 CD36 deficiency regulates cell cycle arrest in the G0/G1 phase via the cyclin D1/CDK4 pathway

**FIGURE 5:**
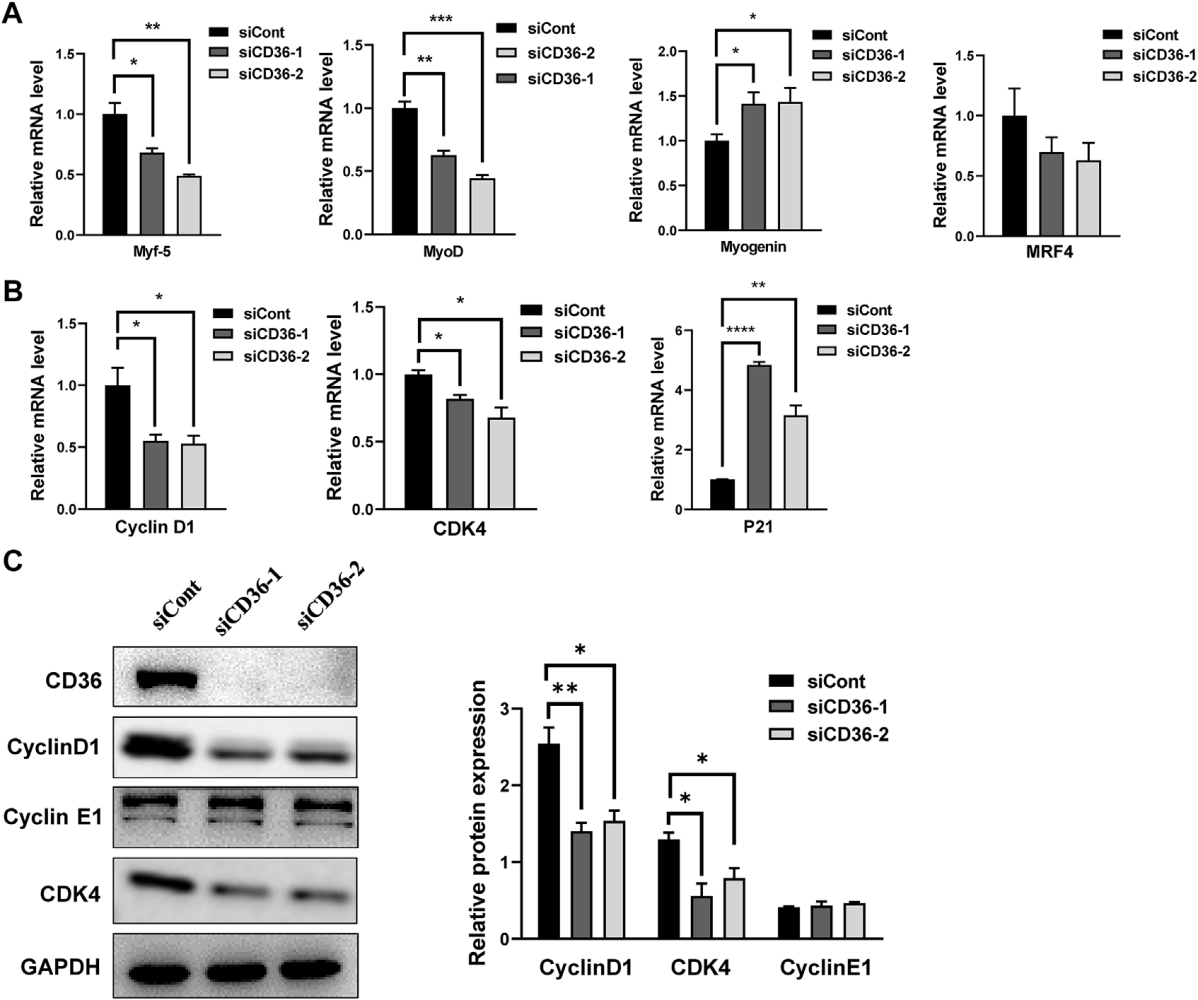
CD36 deficiency regulates cell cycle arrest in G0/G1 phase via the cyclin D1/CDK4 pathway. **(A)** Relative mRNA expression levels of myogenic transcription factors (i.e., Myf5, MyoD, MRF4, and myogenin) in response to CD36 siRNA. **(B)** The expression levels of Cyclin D1, CDK4, and P21 mRNA in C2C12 myoblasts after CD36 knockdown with siRNA. **(C)** Left: the expression levels of CD36, cyclin D1, cyclin E1, and CDK4 protein in C2C12 myoblasts after CD36 siRNA transfection; Right: the quantification of target protein normalized with the control of GAPDH band. Values are expressed as mean ± SEM of three independent experiments. *p < 0.05, **p < 0.01, ***p < 0.001, ****p < 0.0001 vs. the siCont group.

Considering that CD36 loss causes G0/G1 phase arrest, we propose that CD36-mediated signaling pathways may contribute to regulate the cell cycle-related molecule expressions in skeletal muscle cells. Because the formation of multinucleated myotubes is coordinated by myogenic transcription factors, such as myogenin, Myf5, MRF4, and MyoD (Yin et al., 2018), we investigated the effect of CD36 knockdown on the expressions of myogenic transcription factors (i.e., Myf5, MyoD, muscle regulatory factors [MRF] 4, and myogenin) in C2C12 cells. MyoD and Myf5 are found to enhance the muscle progenitor population growth (Megeney et al., 1996; Ustanina et al., 2007). Conversely, myogenin in proliferating myoblasts causes quitting the cell cycle (Liu et al., 2012). Our results showed that CD36 deficiency could trigger the downregulation of Myf5 (*p* < 0.05, *p* < 0.01) and MyoD (*p* < 0.01, *p* < 0.001) and the upregulation of myogenin (*p* < 0.05, *p* < 0.05). This phenomenon indicated that CD36 was critical for the regulation of myogenic transcription factors, thus inhibiting cell cycle progression (Figure 5A). Proliferative markers cyclin D1 (*p* < 0.05, *p* < 0.05) and CDK4 (*p* < 0.05, *p* < 0.05) had downregulated mRNA expression, and CD36 siRNA upregulated the cyclin-dependent kinase inhibitor p21 (*p* < 0.0001, *p* < 0.01; Figure 5B). Furthermore, Western blot analysis was used to detect the expressions of cell cyclin proteins. Results showed that the CD36 knockdown group had reduced Cyclin D1 protein expression compared with the siCont group. CDK4, a protein bound to cyclin D1 to coregulate the cell cycle, was also remarkably reduced (*p* < 0.01, *p* < 0.05; Figure 5C). This finding is consistent with our results above, in which CD36 deletion induced an arrest of the cell cycle in the G0/G1 phase. Collectively, these findings implied that CD36 knockdown probably caused cell cycle arrest in the G0/G1 phase via the cyclin D1/CDK4 pathway.

## 4 Discussion

Dysregulated proliferation of skeletal muscle cells is an important factor in obesity-related muscle atrophy. CD36 is widely expressed in skeletal muscle tissue and plays a critical role in regulating the differentiation process; however, the role of CD36 in skeletal muscle cell proliferation has not been clarified. In this study, PA was used to mimic the HFD model *in vitro*. CD36 was identified as a novel target to determine muscle cell proliferation. Mechanically, the silencing of CD36 promoted cell cycle arrest in the G0/G1 phase by cyclin D1/CDK4, which inhibited skeletal muscle cell proliferation. These studies suggest that targeting CD36 might be a potential therapeutic strategy to protect muscle wasting from obesity.

Mice fed with HFD exhibited a significant decrease in skeletal muscle cell proliferation in skeletal muscle tissue compared to normal diet-fed controls (Hu et al., 2010). Consistent with our *in vivo* findings, the results *in vitro* also demonstrated that PA inhibited the viability of skeletal muscle cells. Similarly, others also reported that PA reduced the viability of rat cardiomyocytes (Yang et al., 2019). Currently, most studies support that PA suppresses cell proliferation and causes apoptosis in various cell types and cell lines, including stem cells (Yuan et al., 2013), hepatocytes (Yin et al., 2018), and neurons (Khullar et al., 2010). However, some study showed that unsaturated FAs, such as OA, promote the proliferation of C2C12 cells (Meng et al., 2018). Presumably, the distinct effects of PA and OA on cell proliferation may be related to the differences in fatty acid composition, cell types, and exposure time. The possible mechanism of the inhibitory effect of PA on the skeletal muscle cell proliferation was further explored.

CD36 takes a critical part in the LCFA sensing, transfer, and metabolism. In this study, CD36 was knocked down with CD36 siRNA to elucidate the role of CD36 in PA-inhibited skeletal muscle cell proliferation. Our results showed that CD36 deficiency further enhanced PA-induced inhibition of C2C12 cell proliferation and promoted apoptosis. RNA-seq analysis was further used to detect the transcriptomic changes of C2C12 cells with and without CD36 expression. Interestingly, genes involved in the cell cycle pathway were highly enriched, and qRT-PCR analysis confirmed the findings. Thus, CD36 may contribute to the proliferation of skeletal muscle cells through cell cycle control. CD36 overexpression has been reported to inhibit cell cycle arrest at the G0/G1 phase in lung cancer cells and promotes lung cancer progression; and CD36 inhibitors have a significant inhibitory effect on the growth of lung tumors (Sun et al., 2018). However, the effect of CD36 expression levels on the skeletal muscle cell cycle is unknown. Cell cycle analysis in our study revealed that CD36 deficiency induced a decrease in population at S phase and an increase in population at G0/G1phase in C2C12 cells, which was consistent with previous study in lung cancer cells (Sun et al., 2018). Together, these observations suggested that CD36 deficiency inhibited skeletal muscle cell proliferation by enhancing G0/G1-phase arrest. Furthermore, our RNA-seq analysis showed that knockdown of CD36 resulted in downregulation of the expression of genes associated with the “dilated cardiomyopathy”, “hypertrophic cardiomyopathy (HCM)” and “ cardiac muscle contraction” pathways, such as Myh6, Myh7, Tnni3 and Cacng6, in skeletal muscle cells, suggesting that CD36 may have an important regulatory role on skeletal muscle gene expression. However, there is a lack of reports on the effects of CD36 on muscle components, especially on skeletal muscle cells, and only a few reports on CD36 deficiency in hypertrophic cardiomyopathy, yet the conclusions remain controversial. Some studies suggested that CD36 deficiency might be an etiological factor in HCM (Tanaka et al., 1997); while others found that CD36 deficiency was not a characteristic factor in HCM and had little impact on the pathyphysiology of HCM (Nakamura et al., 1999). Therefore, we hypothesized that CD36 might play an important regulatory role in skeletal muscle regeneration by regulating skeletal muscle expression genes in addition to increasing skeletal muscle cell proliferation.

Withdrawal from the cell cycle and differentiation into myotubes are regulated by MRFs that including MRF4, myogenin, Myf5, and MyoD (Zammit, 2017). Further studies revealed that MRFs play an important role in bringing precursor cells into myogenic cells and triggering terminal differentiation of myogenic cells (Hernández-Hernández et al., 2017). Studies from cultured cell systems provide evidences for the view that Myf5 and MyoD contribute to the expansion of the muscle progenitor cell pool (Yi et al., 2017), while myogenin induces cell cycle exit. Combined with our findings, we propose that CD36 deficiency may block cell cycle progression through downregulation of MyoD and Myf5 and induce cell cycle exit through upregulation of myogenin. However, the subtle differences in the contribution of various myogenic transcription factors to cell cycle progression in the absence of CD36 should be further explored.

In addition, the coordination of myoblast proliferation and differentiation requires the downregulation of cell cycle activators, such as cyclins and CDKs, and the upregulation of cell cycle inhibitors, such as p21 (De Falco and De Luca, 2006). A group of enzymes known as cyclins and CDKs governs cell cycle regulation to ensure proper DNA replication and chromosomal segregation (Bendris et al., 2015). Different cyclins are required at different stages of the cell cycle. In a variety of cell types, cyclin D1 contributes to the progression of the G0/G1 phase through the activation of CDK4 (Ewen and Lamb, 2004). Cyclin D1/CDK4 pathway causes G1/S-phase conversion, and this pathway is generally defective in many human diseases (Bhowmick et al., 2014). Our findings suggested a significant reduction in cyclin D1 and CDK4 expression levels by the downregulation of CD36 in C2C12 myocytes, which can prolong the G1 phase and inhibit cell proliferation. Thus, this phenomenon may be one of the important molecular mechanisms underlying impaired skeletal muscle cell proliferation in metabolic diseases. Taken together, our study showed that the knockdown of CD36 in C2C12 myoblasts delayed cell cycle progression by blocking the expression of myogenic factors and cell cycle regulators, implying its importance in myoblast proliferation and differentiation. In the future, the mechanism of CD36 involvement in myoblast fusion during myogenic differentiation can be further explored, thus providing new therapeutic targets for skeletal muscle regeneration.

In conclusion, the silencing of CD36 decreased skeletal muscle cell proliferation and promoted apoptosis in an *in vitro* model of PA-treated skeletal muscle cells. Mechanistically, the silencing of CD36 suppressed cell proliferation by preventing the cell cycle from the G0/G1 phase to S phase in cyclin D1/CDK4-dependent manner. This study provides a new understanding of the pathogenesis of obesity-associated muscle wasting, which lays a theoretical foundation for the follow-up mechanism research.

## Data Availability

The datasets presented in this study can be found in online repositories. The names of the repository/repositories and accession number(s) can be found below: https://www.ncbi.nlm.nih.gov/, GSE204686; GSM6185841-GSM6185846.
