# Supplementary material for: CD36 deficiency inhibits proliferation by cell cycle control in skeletal muscle cells
Source: Front Physiol. 2022 Aug 30;13:947325. doi: 10.3389/fphys.2022.947325 (PMC9468905; doi:10.3389/fphys.2022.947325)
Supplement: Supplementary file 1 [file Table1.docx]

**Supplementary Table S1.**

**The list of differentially expressed genes (DEG) between siCont and siCD36 groups**
